# Hematological and blood chemistry profiles of the Mediterranean pond turtle (*Mauremys leprosa*) in a Portuguese wildlife rehabilitation center

**DOI:** 10.3389/fvets.2025.1504336

**Published:** 2025-04-17

**Authors:** Gonçalo N. Marques, Joana S. Guerra, Miriam O. Leal, João Neves

**Affiliations:** ^1^Department of Veterinary Medicine, Zoomarine, Guia, Portugal; ^2^MARE-ISPA – Marine and Environmental Sciences Centre, Lisbon, Portugal; ^3^ARNET – Aquatic Sciences Network, Lisbon, Portugal; ^4^Department of Science and Conservation, Zoomarine, Guia, Portugal

**Keywords:** Mediterranean pond turtle, *Mauremys leprosa*, hematology, blood chemistry, rehabilitation center

## Abstract

The Mediterranean pond turtle (*Mauremys leprosa*) is increasingly recognized as a sentinel species for freshwater ecosystem health due to its resilience to environmental pollutants. Despite its ecological significance, there is a lack of research on the blood profile of this vulnerable species. This study assessed the hematological and biochemical profile of healthy individuals admitted to *Zoomarine*’s *Porto d’Abrigo* rehabilitation center in southern Portugal between 2015 and 2024. This investigation revealed significant differences between the established data and the reference intervals reported in a Spanish study, notably in total erythrocyte (RBC) counts, packed cell volume (PCV), differential counts of lymphocytes and eosinophils, total protein (TP), creatine kinase (CK), potassium (K^+^), phosphorous (PHOS), and glucose (GLU) levels. Significant differences were also observed between captive and wild-rescued individuals in monocyte percentages, aspartate aminotransferase (AST), and CK levels. No seasonal influence was detected except in the differential count of eosinophils. These findings indicate a possible geographical, environmental, and captivity-induced variation, providing the first comprehensive species-specific hematological and biochemical intervals for *M. leprosa* derived from a healthy population. This study enhances the understanding of *M. leprosa* health within a One Health framework by offering critical diagnostic benchmarks for veterinary care and facilitating conservation strategies.

## 1 Introduction

The Mediterranean pond turtle (*Mauremys leprosa*) is a small to medium-sized freshwater turtle, distributed in North Africa, the Iberian Peninsula, and southern France (1). It can be found inhabiting mix-type water bodies, including ponds, streams, marshes, mud banks, swampy meadows, as well as anthropogenic habitats, such as agriculture irrigation systems (1–4). *M. leprosa* coexists with *Emys orbicularis* in some aquatic habitats but appears to be less selective in its habitat requirements (2, 3, 5, 6).

*M. leprosa* was listed as Near Threatened by the International Union for the Conservation of Nature (IUCN) Red List of Threatened Species (7). The distribution area of *M. leprosa* has been significantly reduced, with the decline or even extinction of certain populations, including in Portuguese territory (2, 4, 8). In Spain, it is estimated a loss of 30% of the population in a 60-year period (3). The main threats include habitat fragmentation, water pollution, predation, competition with the invasive species *Trachemys scripta,* and intentional capture for the pet trade (1).

Increased human activity and agricultural practices have greatly contributed to the further degradation of freshwater courses, with the most significant sources of pollution including effluent discharges, agricultural fertilizers and pesticides, hormones, and antibiotics (9, 10). As *M. leprosa* is frequently recorded in highly polluted waters and can resist extremely degraded environments, it has increasing potential as a biological sentinel species in a One Health framework, providing insights into the overall health of freshwater ecosystems (11, 12). Monitoring the health status of these long-lived turtles and integrating them into a One Health approach could potentially aid in the early detection of emerging threats, especially in human-shared environments, as they can easily be exposed to infectious agents and anthropogenic environmental contaminants (11, 13).

Although some research has been conducted on *M. leprosa*, the literature remains limited. Notably, a comprehensive study by Hidalgo-Vila et al. (14) examined hematological and biochemical reference intervals (RI) in a wild-caught Spanish population of 114 individuals. Hematology works are established as a vital diagnostic tool in the comprehensive health assessment of reptile species, providing valuable diagnostic evidence on disease detection and monitoring. However, even though reptile hematology and blood chemistry have advanced greatly throughout the last decades, the interpretation of reptilian hematologic results is still anecdotal and frequently based on mammalian assumptions (15). Further investigation is still needed, for example, when it concerns unknown or imprecise RI of some reptile species.

Population-based RI are one of the most common tools used diagnostic-wise, although criteria standards are essential to produce validated and robust RI (16). The American Society of Veterinary Clinical Pathology (ASVCP) established guidelines on the determination of RI, including a thorough selection of the reference individuals through specific exclusion criteria (16). Only after establishing reptile species-specific RI, it is possible to assess any value deviation, and further explore possible causes influencing both the overall health status and welfare of these animals.

It was the authors’ purpose to carry out a study on the hematological and blood chemistry profiles of *M. leprosa* individuals admitted to *Zoomarine*’s wildlife rehabilitation center *Porto d’Abrigo*, offering baseline data for a practical medical evaluation of the population of *M. leprosa* based in southern Portugal. Furthermore, the authors intended to investigate the differences between the blood profiles here reported and the ones described in a Spanish population by Hidalgo-Vila et al. (14).

*Porto d’Abrigo* admits not only rescued terrapins showing external signs of illness and/or found in urban centers or other locations isolated from nearby ponds or streams but also individuals who were kept under human care as pets. These animals undergo an evidence-based medical and behavioral rehabilitation process before they are released to the wild. As such, this study also aimed to explore the impact of captivity on hematological and biochemical data, as well as the influence of seasonality on the blood profile of *M. leprosa*. To the authors’ best knowledge, this is the first study to investigate the hematological and biochemical profiles of *M. leprosa* in Portugal and the first worldwide based on healthy individuals from a wildlife rehabilitation center.

## 2 Materials and methods

This retrospective study was carried out at *Zoomarine*’s *Porto d’Abrigo*, an aquatic wildlife rehabilitation center in Algarve, southern Portugal. All *M. leprosa* individuals admitted to *Porto d’Abrigo* between September 2015 and July 2024 were considered. Exclusion criteria were early defined and only healthy specimens were enrolled in the study. Individuals were deemed healthy if they had an unremarkable physical examination, no clinical signs, and no changes on the routine diagnostic exams (i.e., radiography, coprological examination).

All animal procedures were carried out by veterinarians, veterinarian nurses, or other qualified auxiliary personnel. Blood sampling and other diagnostic techniques were part of the routine procedure at the entrance of the terrapins in the rehabilitation center or for diagnostic and monitoring purposes. No procedure was performed purposefully for this retrospective study.

### 2.1 Sample collection

Blood samples were obtained from the jugular vein with 23-or 25-gauge needles and 1-or 2-mL syringes, according to the size of the terrapin. Blood samples were immediately transferred into lithium heparin tubes and processed the same day. A blood smear was performed using a remaining drop of blood from the syringe, avoiding contact with the anticoagulant. The animals were physically restrained for this procedure, and no sedation or anesthesia was required.

### 2.2 Sample processing

Blood samples were analyzed for an in-house hemogram and biochemistry panel. Both total erythrocyte (RBC, × 10^6^/μL) and leukocyte (WBC, × 10^3^/μL) counts were done manually. A 5 μL blood-filled pipette was inserted into a Natt-Herricks-TIC® 1:200 stain solution vial, and counting was performed with a Neubauer chamber. Hemoglobin (Hb – g/dL) levels were measured with a hemoglobin analyzer (HemoCue®) and packed cell volume (PCV – %) was determined after centrifugation of microhematocrit tubes (Centurion Scientific Ltd. - Pro-Vet, 12,000 rpm, 5 min). Blood smears were evaluated following Diff-Quik staining. The biochemical profile was analyzed using a VETSCAN® VS chemistry analyzer (Avian/Reptilian Profile Plus), in which the following parameters were determined: aspartate aminotransferase (AST – U/L), bile acids (BA – μmol/L), creatine kinase (CK – U/L), uric acid (UA – mg/dL), glucose (GLU – mg/dL), Phosphorous (PHOS – mg/dL), Calcium (Ca^2+^ − mg/dL), Total Protein (TP – g/dL), Albumin (ALB – g/dL), Globulins (GLOB – g/dL), Potassium (K^+^ − mmol/L) and Sodium (Na^+^ − mmol/L).

### 2.3 Data collection and statistical analysis

By reviewing the medical files of the 100 terrapins admitted to *Porto d’Abrigo* (2015–2024), 23 healthy individuals were identified, thus 77 animals were excluded due to any deviation compatible with the exclusion criteria or due to unavailable information. Hematological and biochemical data from the 23 healthy terrapins were compiled into an Excel spreadsheet.

If an animal had more than one blood register, only the first sample was considered. The date (day, month, year) of the blood collection was registered. Nine individuals were kept as pets before being received by the rehabilitation center, while the remaining terrapins were wild-rescued.

The intervals for the 20 hematological and biochemical parameters were established based on data from the 23 healthy individuals. For statistical purposes, values exceeding the detection limits of the VETSCAN® VS chemistry analyzer for Ca^2+^ (>16 mg/dL) and K^+^ (>8 mmol/L) were excluded – 2/23 and 1/23 cases, respectively.

Statistical analyses (descriptive and inferential) were conducted using the SPSS program (version 26). Descriptive statistics included standard deviation (SD), minimum (Min), maximum (Max), lower reference limit (LRL), upper reference limit (URL), and 90% confidence intervals (CI) for all hematological and biochemical parameters measured. Cohen’s d was calculated to measure effect size when comparing the Portuguese and Spanish populations. Values of 0.2, 0.5, and 0.8 indicated small, medium, and large effect sizes, respectively, allowing assessment of practical significance of the observed differences beyond statistical significance.

To investigate variations between the data obtained from the terrapins at *Porto d’Abrigo* with the RI described in the Spanish population by Hidalgo-Vila et al. (14), an independent *t*-test was used. The comparison was performed using the mean RI for females and males described in the Spanish study. A comparison of Hb, ALB, and GLOB values was not feasible since these parameters were not assessed in the Spanish population. Moreover, AST levels and basophil counts were not compared due to the divergence of measures, as Hidalgo-Vila et al. defined RI by the median rather than the mean.

To evaluate the influence of seasonality, animals were separated into two groups, according to the month of the blood sample collection – the Winter group comprehended the terrapins sampled between November and April, while the Summer group included the individuals sampled between May and October.

To investigate the influence of captivity and seasonality within the collected data, as it required the breaking down of data into smaller samples, a Shapiro–Wilk test was performed to assess normal distribution. As most data did not follow a normal distribution, a Mann–Whitney *U* test was performed to check for differences between conditions (Wild vs. Captive; Summer vs. Winter).

## 3 Results

The intervals for the evaluated parameters in the healthy *M. leprosa* are presented in Table 1. BA levels were consistently below 35 μmol/L.

**Table 1:** Hematological and biochemical profiles of the healthy *Mauremys leprosa* admitted to *Porto d’Abrigo*, a wildlife rehabilitation center.

Parameters	*N*	Mean ± SD	Median	Min-Max	LRL 90% CI	URL 90% CI
RBC (× 10^6^/μL)	21	0.60 ± 0.14	0.60	0.35–0.84	0.32–0.39	0.76–0.92
Hb (g/dL)	23	6.86 ± 1.47	7.20	3.80–10.20	3.42–4.18	9.18–11.22
PCV (%)	23	26.94 ± 7.12	27.00	16.00–50.00	14.40–17.60	45.00–55.00
WBC (× 10^3^/μL)	21	3.93 ± 1.32	3.67	1.56–6.00	1.40–1.71	5.40–6.60
Heterophils (%)	20	61.53 ± 15.33	61.45	32.30–86.70	29.07–35.53	78.03–95.37
Eosinophils (%)	20	4.97 ± 4.50	3.90	0.00–17.00	0	15.30–18.70
Basophils (%)	20	0.23 ± 0.65	0.0	0.00–2.70	0	2.43–2.97
Lymphocytes (%)	20	28.89 ± 13.73	31.20	2.90–57.90	2.61–3.19	52.11–63.69
Monocytes (%)	20	3.19 ± 2.34	3.80	0.00–8.00	0	7.20–8.80
UA (mg/dL)	21	1.31 ± 0.74	1.20	0.20–3.00	0.18–0.22	2.70–3.30
TP (g/dL)	21	4.76 ± 1.43	4.50	1.90–7.80	1.71–2.09	7.02–8.58
ALB (g/dL)	20	1.80 ± 0.65	1.80	0.70–3.30	0.63–0.77	2.97–3.63
GLOB (g/dL)	20	3.11 ± 0.77	2.90	1.80–4.80	1.62–1.98	4.32–5.28
GLU (mg/dL)	21	49.38 ± 22.01	46.00	12.00–122.00	10.80–13.20	109.80–134.20
AST (U/L)	21	276.19 ± 227.61	215.00	85.00–996.00	76.50–93.50	896.40–1095.60
CK (U/L)	21	2317.33 ± 1802.82	1710.00	569.00–7240.00	512.10–625.90	6516.00–7964.00
Na^+^ (mmol/L)	21	130.62 ± 8.86	131.00	110.00–142.00	99.00–121.00	127.80–156.20
K^+^ (mmol/L)	20	5.90 ± 1.07	5.40	4.70–8.50	4.23–5.17	7.65–9.35
Ca^2+^ (mg/dL)	19	11.72 ± 2.53	11.00	8.80–16.90	7.92–9.68	15.21–18.59
PHOS (mg/dL)	21	4.17 ± 1.28	4.10	2.30–7.00	2.07–2.53	6.30–7.70

N, number of individuals; SD, standard deviation; Min, minimum; Max, maximum; LRL, lower reference limit; URL, upper reference limit; CI, confidence interval; RBC, erythrocytes; Hb, hemoglobin; PCV, packed cell volume; WBC, leukocytes; UA, uric acid; TP, total protein; ALB, albumin; GLOB, globulins; GLU, glucose; AST, aspartate aminotransferase; CK, creatine kinase; Na^+^, sodium; K^+^, potassium; Ca^2+^, calcium; PHOS, phosphorous.

Table 2 compares the hematological and biochemical profiles of the terrapins admitted to *Porto d’Abrigo* with those of the captured terrapins by Hidalgo-Vila et al. (*α* = 0.05). Hematological statistical differences were found in the total RBC count, PCV, and differential WBC counts of lymphocytes and eosinophils – all higher in the Portuguese individuals except for the percentage of eosinophils. Biochemical differences included higher levels of TP, CK, K^+^, and PHOS in the Portuguese individuals, while GLU levels were higher in the Spanish population.

**Table 2:** Comparison between the hematological and biochemical profiles of *Mauremys leprosa* admitted to *Porto d’Abrigo* with those of the captured *M. leprosa* by Hidalgo-Vila et al. (14).

Parameters	*N*	Mean ± SD PT population	*N*	Mean/Median ± SD ES population	*t*	df	*p*-value	Cohen’s d
RBC (× 10^6^/μL)	21	0.60 ± 0.14*	111	0.37 ± 0.17	6.25	130	< 0.01	1.38
PCV (%)	23	26.94 ± 7.12*	111	19.45 ± 4.97	6.48	132	< 0.01	1.39
WBC (× 10^3^/μL)	21	3.93 ± 1.32	60	4.49 ± 2.43	−1.03	79	0.30	−0.26
Heterophils (%)	20	61.53 ± 15.33	55	56.28 ± 17.21	1.21	73	0.23	0.32
Eosinophils (%)	20	4.97 ± 4.50*	55	33.88 ± 17.07	−7.47	73	< 0.01	−1.80
Lymphocytes (%)	20	28.89 ± 13.73*	55	6.04 ± 3.92	11.54	73	< 0.01	2.94
Monocytes (%)	20	3.19 ± 2.34	55	3.09	-	-	-	-
UA (mg/dL)	21	1.31 ± 0.74	111	1.55 ± 1.01	−1.06	130	0.29	−0.25
TP (g/dL)	21	4.76 ± 1.43*	109	3.25 ± 1.30	4.85	128	< 0.01	1.13
GLU (mg/dL)	21	49.38 ± 22.01*	109	73.86 ± 37.83	−2.91	128	< 0.01	−0.71
CK (U/L)	21	2317.33 ± 1802.82*	110	1580.69 ± 1064.62	2.51	129	0.01	0.58
Na^+^ (mmol / L)	21	130.62 ± 8.86	107	132.45 ± 3.21	−1.50	126	0.14	−0.34
K^+^ (mmol / L)	20	5.90 ± 1.07*	108	3.40 ± 0.45	15.98	126	< 0.01	3.57
Ca^2+^ (mg/dL)	19	11.72 ± 2.53	111	12.06 ± 3.61	−0.39	128	0.70	−0.10
PHOS (mg/dL)	21	4.17 ± 1.28*	112	2.79 ± 1.24	4.71	131	< 0.01	1.10

*N*, number of individuals; SD, standard deviation; PT, portuguese; ES, spanish; *t*, t-test statistic value; df, degrees of freedom; * significant diferences; RBC, erythrocytes; PCV, packed cell volume; WBC, leukocytes; UA, uric acid; TP, total protein; GLU, glucose; CK, creatine kinase; Na^+^, sodium; K^+^, potassium; Ca^2+^, calcium; PHOS, phosphorous.

Table 3 refers to the statistical comparison of the blood parameters between the 9 captive animals (C) and the 14 animals rescued from the wild (W). The only significant hematological difference between the different origin groups was in the percentage of monocytes, which was higher in the terrapins of wild origin (*U* = 81.50, *p* = 0.02). There were also significant differences found between groups in two biochemical parameters: AST (*U* = 19.00, *p* = 0.02) and CK (*U* = 22.00, *p* = 0.03), both higher in the wild-rescued individuals.

**Table 3:** Influence of origin – Wild (W) vs. Captive (C) – on the hematological and biochemical profiles of *Mauremys leprosa*.

Parameters	Origin	*N*	Mean ± SD	Min-Max	LRL 90% CI	URL 90% CI
RBC (× 10^6^/μL)	W	12	0.59 ± 0.15	0.35–0.84	0.32–0.39	0.76–0.92
	C	9	0.62 ± 0.13	0.42–0.80	0.38–0.46	0.72–0.88
Hb (g/dL)	W	14	6.46 ± 1.44	3.80–8.60	3.42–4.18	7.74–9.46
	C	9	7.47 ± 1.37	5.20–10.20	4.68–5.72	9.18–11.22
PCV (%)	W	14	26.39 ± 8.39	16.00–50.00	14.40–17.60	45.00–55.00
	C	9	27.78 ± 4.89	18.00–35.00	16.20–19.80	31.50–38.50
WBC (× 10^3^/μL)	W	12	4.09 ± 1.51	1.56–6.00	1.40–1.71	5.40–6.60
	C	9	3.72 ± 1.07	2.00–5.33	1.80–2.20	4.80–5.87
Heterophils (%)	W	11	56.72 ± 15.18	32.30–86.70	29.07–35.53	78.03–95.37
	C	9	67.41 ± 14.11	45.00–84.40	40.50–49.50	75.96–92.84
Eosinophils (%)	W	11	4.62 ± 3.94	0.00–14.80	0.00–0.00	13.32–16.28
	C	9	5.40 ± 5.31	0.80–17.00	0.72–0.88	15.30–18.70
Basophils (%)	W	11	0.32 ± 0.83	0.00–2.70	0.00–0.00	2.43–2.97
	C	9	0.11 ± 0.33	0.00–1.00	0.00–0.00	0.90–1.10
Lymphocytes (%)	W	11	31.66 ± 16.62	2.90–57.90	2.61–3.19	52.11–63.69
	C	9	25.51 ± 8.91	16.00–40.00	14.40–17.60	36.00–44.00
Monocytes (%)	W	11	4.33 ± 2.38*	0.00–8.00	0.00–0.00	7.20–8.80
	C	9	1.79 ± 1.39*	0.00–4.00	0.00–0.00	3.60–4.40
UA (mg/dL)	W	13	1.53 ± 0.67	0.80–3.00	0.72–0.88	2.70–3.30
	C	8	0.95 ± 0.73	0.20–2.20	0.18–0.22	1.98–2.42
TP (g/dL)	W	13	4.32 ± 1.32	1.90–6.80	1.71–2.09	6.12–7.48
	C	8	5.49 ± 1.36	4.20–7.80	3.78–4.62	7.02–8.58
ALB (g/dL)	W	12	1.60 ± 0.63	0.70–2.80	0.63–0.77	2.52–3.08
	C	8	2.09 ± 0.61	1.30–3.30	1.17–1.43	2.97–3.63
GLOB (g/dL)	W	12	2.93 ± 0.62	1.80–4.00	1.62–1.98	3.60–4.40
	C	8	3.38 ± 0.94	2.20–4.80	1.98–2.42	4.32–5.28
GLU (mg/dL)	W	13	51.69 ± 26.16	12.00–122.00	10.80–13.20	109.80–134.20
	C	8	45.63 ± 13.58	27.00–66.00	24.30–29.70	59.40–72.60
AST (U/L)	W	13	342.62 ± 254.80*	119.00–996.00	107.10–130.90	896.40–1095.60
	C	8	168.25 ± 123.38*	85.00–436.00	76.50–93.50	392.40–479.60
CK (U/L)	W	13	2891.69 ± 2079.66*	936.00–7240.00	842.40–1029.60	6516.00–7964.00
	C	8	1384.00 ± 513.49*	569.00–2284.00	512.10–625.90	2055.60–2512.40
Na^+^ (mmol/L)	W	13	131.39 ± 8.52	115.00–142.00	103.50–126.50	127.80–156.20
	C	8	129.38 ± 9.84	110.00–140.00	99.00–121.00	126.00–154.00
K^+^ (mmol/L)	W	13	6.02 ± 1.26	4.70–8.50	4.23–5.17	7.65–9.35
	C	7	5.67 ± 0.59	5.00–6.50	4.50–5.50	5.85–7.15
Ca^2+^ (mg/dL)	W	11	10.88 ± 2.35	8.80–15.90	7.92–9.68	14.31–17.49
	C	8	12.86 ± 2.46	9.30–16.90	8.37–10.23	15.21–18.59
PHOS (mg/dL)	W	13	4.10 ± 1.45	2.30–7.00	2.07–2.53	6.30–7.70
	C	8	4.28 ± 1.02	2.50–5.80	2.25–2.75	5.22–6.38

*N*, number of individuals; SD, standard deviation; Min, minimum; Max, maximum; LRL, lower reference limit; URL, upper reference limit; CI, confidence interval; W, wild; C, captive; * significant differences; RBC, erythrocytes; Hb, hemoglobin; PCV, packed cell volume; WBC, leukocytes; UA, uric acid; TP, total protein; ALB, albumin; GLOB, globulins; GLU, glucose; AST, aspartate aminotransferase; CK, creatine kinase; Na^+^, sodium; K^+^, potassium; Ca^2+^, calcium; PHOS, phosphorous.

The influence of seasonality is detailed in Table 4. Among the parameters examined, only the differential count of eosinophils showed significant differences between the Winter (Wi) and Summer (S) groups, the latter displaying higher values (*U* = 20.50, *p* = 0.03).

**Table 4:** Influence of seasonality on the hematological and biochemical profiles of *Mauremys leprosa* sampled in colder months [Winter (Wi) group, between November and April] and in warmer months [Summer (S) group – between May and October].

Parameters	Season	*N*	Mean ± SD	Min-Max	LRL 90% CI	URL 90% CI
RBC (× 10^6^/μL)	S	13	0.58 ± 0.14	0.35–0.80	0.32–0.39	0.72–0.88
	W	8	0.65 ± 0.13	0.47–0.84	0.42–0.52	0.76–0.92
Hb (g/dL)	S	15	6.80 ± 1.65	3.80–10.20	3.42–4.18	9.18–11.22
	W	8	6.96 ± 1.13	4.80–8.20	4.32–5.28	7.38–9.02
PCV (%)	S	15	27.40 ± 8.33	16.00–50.00	14.40–17.60	45.00–55.00
	W	8	26.06 ± 4.40	17.00–30.00	15.30–18.70	27.00–33.00
WBC (× 10^3^/μL)	S	13	3.83 ± 1.20	2.00–5.67	1.80–2.20	5.10–6.23
	W	8	4.10 ± 1.57	1.56–6.00	1.40–1.71	5.40–6.60
Heterophils (%)	S	12	62.07 ± 17.41	32.30–86.70	29.07–35.53	78.03–95.37
	W	8	60.73 ± 12.65	46.00–78.90	41.40–50.60	71.01–86.79
Eosinophils (%)	S	12	6.56 ± 5.07*	0.80–17.00	0.72–0.88	15.30–18.70
	W	8	2.59 ± 1.90*	0.00–5.40	0.00–0.00	4.86–5.94
Basophils (%)	S	12	0.08 ± 0.29	0.00–1.00	0.00–0.00	0.90–1.10
	W	8	0.44 ± 0.96	0.00–2.70	0.00–0.00	2.43–2.97
Lymphocytes (%)	S	12	28.30 ± 13.58	7.30–57.90	6.57–8.03	52.11–63.69
	W	8	29.78 ± 14.86	2.90–46.00	2.61–3.19	41.40–50.60
Monocytes (%)	S	12	3.17 ± 2.51	0.00–8.00	0.00–0.00	7.20–8.80
	W	8	3.21 ± 2.23	0.00–5.40	0.00–0.00	4.86–5.94
UA (mg/dL)	S	14	1.21 ± 0.76	0.20–3.00	0.18–0.22	2.70–3.30
	W	7	1.51 ± 0.69	0.50–2.40	0.45–0.55	2.16–2.64
TP (g/dL)	S	14	4.64 ± 1.30	1.90–6.80	1.71–2.09	6.12–7.48
	W	7	5.00 ± 1.73	3.20–7.80	2.88–3.52	7.02–8.58
ALB (g/dL)	S	13	1.82 ± 0.60	0.70–2.80	0.63–0.77	2.52–3.08
	W	7	1.74 ± 0.80	0.90–3.30	0.81–0.99	2.97–3.63
GLOB (g/dL)	S	13	3.02 ± 0.62	2.20–4.00	1.98–2.42	3.60–4.40
	W	7	3.27 ± 1.03	1.80–4.80	1.62–1.98	4.32–5.28
GLU (mg/dL)	S	14	43.21 ± 14.35	12.00–67.00	10.80–13.20	60.3–73.7
	W	7	61.71 ± 30.03	30.00–122.00	27.00–33.00	109.80–134.20
AST (U/L)	S	14	261.93 ± 233.92	85.00–996.00	76.50–93.50	896.40–1095.60
	W	7	304.71 ± 229.62	91.00–645.00	81.90–100.10	580.50–709.50
CK (U/L)	S	14	1975.93 ± 1629.98	569.00–7240.00	512.10–625.90	6516.00–7964.00
	W	7	3000.14 ± 2064.33	967.00–7192.00	870.30–1063.70	6472.80–7911.20
Na^+^ (mmol/L)	S	14	131.36 ± 8.49	115.00–142.00	103.50–126.50	127.80–156.20
	W	7	129.14 ± 10.07	110.00–140.00	99.00–121.00	126.00–154.00
K^+^ (mmol/L)	S	13	6.08 ± 1.18	4.70–8.50	4.23–5.17	7.65–9.35
	W	7	5.57 ± 0.81	4.70–7.20	4.23–5.17	6.48–7.92
Ca^2+^ (mg/dL)	S	12	12.04 ± 2.17	9.30–15.90	8.37–10.23	14.31–17.49
	W	7	11.16 ± 3.16	8.80–16.90	7.92–9.68	15.21–18.59
PHOS (mg/dL)	S	14	4.34 ± 1.32	2.50–7.00	2.25–2.75	6.30–7.70
	W	7	3.81 ± 1.20	2.30–5.80	2.07–2.53	5.22–6.38

*N*, number of individuals; SD, standard deviation; Min, minimum; Max, maximum; LRL, lower reference limit; URL, upper reference limit; CI, confidence interval; S, Summer group; Wi, winter group; * significant differences; RBC, erythrocytes; Hb, hemoglobin; PCV, packed cell volume; WBC, leukocytes; UA, uric acid; TP, total protein; ALB, albumin; GLOB, globulins; GLU, glucose; AST, aspartate aminotransferase; CK, creatine kinase; Na^+^, sodium; K^+^, potassium; Ca^2+^, calcium; PHOS, phosphorous.

## 4 Discussion

Climate change events and human action are further altering and reducing the natural habitat of *M. leprosa*, while the lack of research on this species hampers effective conservation efforts (7). Existing hematology and blood chemistry RI for *M. leprosa* are scarce, and there are no published reports of blood profiles in healthy *M. leprosa* from Portugal.

A statistical comparison between the blood profiles described in this study and those reported by Hidalgo-Vila et al. in a Spanish population of terrapins identified significant differences in several blood parameters. The total RBC count, PCV, differential count of lymphocytes, TP, CK, K^+^, and PHOS were all higher in the Portuguese individuals. Contrarily, the eosinophils percentage and GLU levels were significantly higher in the Spanish population. The latter study was based on individuals captured in the Doñana Biological Reserve (Huelva, southwestern Spain), in different periods of their annual cycle. Because the specimens from this Spanish population had not undergone a prior medical evaluation, possible ill animals might have been included in the study, potentially altering the physiological RI.

A closer examination of the magnitude of these differences (Cohen’s d values) revealed large effects in several parameters, particularly lymphocyte percentage (*d* = 2.94) and potassium levels (*d* = 3.57). These pronounced differences suggest physiological divergences between the Portuguese and Spanish *M. leprosa* populations. While methodological variations may contribute to these disparities, the magnitude of difference, particularly in lymphocyte counts, likely reflects genuine biological adaptations to distinct environmental conditions or potential genetic divergence between populations. Although we cannot entirely rule out that the difference in sample size between studies might influence the precision of these specific effect size estimates, the particularly noticeable effect size for lymphocyte percentage merits special consideration. Such pronounced differences in leukocyte profiles between populations may not only reflect methodological variations but also potentially different immune status or responses to environmental factors. To the authors’ knowledge, the genetic variability between Portuguese and Spanish populations of *M. leprosa* has not yet been explored, which could also provide further insights into the differences in the blood profiles of the two populations. While the current findings support the need to establish RI for different environmental and geographical areas, a premise already supported in other, better-studied reptile species, further research is needed to explore the influence of each variable on the blood profile of *M. leprosa* (17).

Regarding the venipuncture site, while Hidalgo-Vila et al. obtained blood samples from the occipital venous sinus, the jugular vein was used as the collection site in the present study. As previously documented, the site of blood collection in reptiles may influence both hematological and biochemical parameters (18). Consistent with the present findings, a study on the effect of the venipuncture site on the blood profile of desert tortoises (*Gopherus agassizii*) showed lower RBC counts and PCV in samples collected from the occipital sinus compared to jugular vein samples, attributing these differences to the hemodilution of the occipital region samples with extravascular fluid and/or lymph (19). Bonnet et al. (20) also described a significant influence of the puncture site on the blood profile of *M. leprosa* in a captured population in Morocco, although the authors did not consider the occipital sinus, focusing instead on the jugular vein, subcarapacial plexus, and the coccygeal plexus.

To ensure a more robust and replicable comparison between blood results it is important to maintain a consistent laboratory hematology approach. However, total WBC and RBC counts were conducted using different methods: while the present study used the Natt and Herrick’s ready-to-use staining method, Hidalgo-Vila et al. employed the methodology of Campbell (1996) (18). Similarly, the biochemical profile in this study was analyzed using the VETSCAN® VS chemistry analyzer (Avian/Reptilian Profile Plus), whereas the Spanish study used the Roche/Hitachi Modular Analytics automated chemistry analyzer. Variations in the venipuncture site and the different laboratory methodologies between studies may introduce potential biases, further explaining the observed differences between the Portuguese and Spanish blood profiles.

As previously reported, and in contrast to many reptiles, the most common leukocytes in *M. leprosa* are not lymphocytes, but rather heterophils (17). This pattern was also observed in the Portuguese population of *M. leprosa*, which showed a mean heterophil differential count of 61.53% (compared to a heterophil mean of 56.28% in the Spanish population) and a mean lymphocyte differential count of 28.89% (compared to a mean of 6.04% in the Spanish population) (17). The significantly low percentage of lymphocytes observed by Hidalgo-Vila et al. might have been influenced by environmental factors, as a study on *Emys orbicularis* inhabiting the same locality presented similar results (21). Conversely, a study by Muñoz and De La Fuente (22) on the lymphoid distribution in the blood, spleen, and thymus of *M. leprosa* reported lymphocytes as the predominant WBC subpopulation in the blood (57.8–75.5%). The timing of blood collection may also be an important variable influencing the heterophil:lymphocyte ratio. However, further conclusions on this matter cannot be drawn, as the study by Hidalgo-Vila et al. does not specify the timing of blood collection after capture. Additionally, the period between the animals’ rescue and the blood collection for the terrapins admitted to *Porto d’Abrigo* is either variable or unknown, as many animals were not rescued by *Porto d’Abrigo*’s personnel.

The eosinophils percentage was considerably higher in the Spanish population (mean of 33.88%) compared to the present findings in the Portuguese population (mean of 4.97%). Although eosinophilia in mammal medicine is commonly associated with parasitic diseases or allergic disorders, this relationship in reptiles is not straightforward, and more complementary studies are needed. Since no coprological studies were performed in the Spanish population at the time, a high parasitic load cannot be ruled out. Interestingly, Hidalgo-Vila et al. reported infection with *Serpinema microcephalus* and *Falcaustra donanaensis* in *M. leprosa* captured in the same biological reserve a few years earlier (2002–2004) (23, 24). Additionally, an erroneous identification of heterophils as eosinophils is possible, given their similar size and shape, though eosinophilic granules are usually spherical, whereas the granules of heterophils are oval (15).

An elevation of CK is generally attributed to muscle injury, including skeletal, smooth muscle, and cardiac myocytes (17). Although the physical examination of the terrapins included in this study was unremarkable, increased CK levels due to stress in recently admitted animals in a rehabilitation center is plausible. Previous research reports that handling can rapidly trigger a significant stress response in *M. leprosa* (20). Higher activities of CK and AST have also been related to animal restraining or holding *per se* in some reptile species (25). Additionally, a recent history of muscle trauma prior to the admittance of the animal cannot be ruled out and might further explain higher values compared to the RI of the Spanish population.

The higher TP and PCV values in the Portuguese terrapins raise the possibility of any subclinical dehydration not evident in the physical examination. Finally, physiological differences of GLU, K^+^, and PHOS are reported in other reptiles, influenced, for example, by metabolic rate, activity levels, and environmental adaptations, with no apparent presence of disease or reproductive activity (26). As the reproduction status may influence the reptile blood profile, no females with eggs detected on the radiographic examination were included in this study. However, reproductive activity cannot be entirely dismissed as the sensitivity of the radiographic exam depends on the degree of eggshell calcification.

Other factors that influence the blood parameters of reptiles include age and sex (14, 15, 17, 20). Age is not determined at the admittance of *M. leprosa* in *Porto d’Abrigo*, due to the lack of morphometric guidelines in this species. Also, the influence of sex on blood parameters was not discussed as only 19 out of 23 individuals had their sex recorded in their medical sheet – 5 males and 14 females – which was not statistically relevant. The absence of sex information was due to unrecorded data in the medical archives. Besides the direct observation of the phallus in males, there are several external characteristics that help sex identification of *M. leprosa*, although they are inherently subjective, unreliable, and especially difficult to evaluate in non-sexual matured individuals (1, 27–29). Hidalgo-Vila et al. described higher values of PCV, RBC, and WBC counts in males (14). Contrarily, all the biochemical values were higher in females, except for lactate dehydrogenase (LDH) and creatinine. Bonnet et al. (20) also found a significant sex effect in some biochemical parameters of *M. leprosa*, including triglycerides, PHOS, and iron levels (20).

The impact of captivity on the blood profile of *M. leprosa* was evaluated by comparing the blood results of the 9 individuals held as pets with the blood profile of the 14 rescued individuals. It is important to note that a recent history of captivity of these 14 wild-rescued individuals cannot be entirely ruled out, as they might have escaped or been abandoned, before being encountered in urban centers or other sites away from a nearby stream. Animals under human care presented lower values of AST and CK, as well as a lower percentage of monocytes in the smear evaluation. Stress levels and captivity status are known to influence blood parameters in reptiles (30). The well-studied mammal stress leukogram is commonly associated with neutrophilia, lymphopenia, eosinopenia, and potentially monocytosis (31). Similarly, increased glucocorticoid levels in reptiles have been linked to heterophilia, lymphopenia, and degranulation of the granulocytes (30). Notwithstanding the increased percentage of monocytes in the wild-origin group as the only significant variation of the WBC differential count, it is interesting to note the higher levels of AST and CK in wild-origin individuals. Although the influence of captivity on chronic stress is a well-studied subject in exotic animals, wild animals often suffer muscle injuries and may be subjected to higher levels of chronic and/or acute stress, especially considering habitat fragmentation, food availability, competition, and other environmental stressors associated to human activity and climate change. Moreover, as the wild-origin individuals had little to no prior human contact, in contrast to their captive counterparts, they may have responded more severely to handling and restraint, leading to increased muscle activity or even muscle damage (20).

Finally, although seasonality and hibernation are described as influencing factors in the physiological RI of reptiles, no significant differences were found between the terrapins admitted to the rehabilitation center in the warmer versus colder months, except for a higher eosinophil percentage in the Summer group (15, 17, 32). In contrast, Muñoz & De La Fuente described no eosinophil seasonal variation in *M. leprosa* males, while females had a higher percentage of eosinophils when captured in colder months (from January 15 to February 20) (22). Pagés et al. (32) also found seasonal changes in two groups of captured *M. leprosa* in Spain. The July group (*N* = 7) presented lower values of PCV, RBC, and Hb, as well as lower plasma concentrations of GLU, Ca^2+^, and magnesium compared to the November group (*N* = 5), which had significantly lower levels of PHOS.

*Porto d’Abrigo* features both indoor and outdoor habitats for terrapins. Upon admission, they are typically housed in indoor habitats with ultraviolet lighting (UVA and UVB). Based on their clinical status, they are then transferred to the outdoor pools. Key water parameters, monitored at least twice daily, include temperature (maintained above 18°C in winter and below 26°C in summer), pH levels, ammonia, and free and total chlorine levels. The lack of significant findings between different seasons in this study may be attributed to the relatively mild winters in the Algarve region, compared to other regions of the Iberian Peninsula. Further comparison of seasonal effects was not possible, as the study by Hidalgo-Vila et al. (14) lacks detailed environmental data, including temperature and water quality parameters.

The main limitation of this research comprehends the small sample size, which, while posing statistical challenges, is a common difficulty in ecological studies involving wild individuals. Given the current sample size, these hematological and biochemical data can only serve as preliminary RI. However, the strict exclusion criteria (i.e., alterations in the physical examination, clinical signs, and/or abnormal findings on the radiographic or coprological examination) ensured more accurate physiological intervals. Considering its ecological significance, and its potential role as a sentinel species for animal, environmental, and human health, future research should involve further geographical comparisons and genetic investigation in larger sample sizes, while also exploring the influences of habitat pollution and fragmentation.

## Data Availability

The datasets analyzed for this study can be made available (hematological and biochemical values of each individual). Requests to access these datasets should be directed to GM, goncalo.marques@zoomarine.pt.
